# Evidence that mutation accumulation does not cause aging in *Saccharomyces cerevisiae*

**DOI:** 10.1111/acel.12290

**Published:** 2015-02-22

**Authors:** Alaattin Kaya, Alexei V Lobanov, Vadim N Gladyshev

**Affiliations:** Division of Genetics, Department of Medicine, Brigham and Women's Hospital and Harvard Medical SchoolBoston, MA, 02115, USA

**Keywords:** aging, DNA damage, lifespan, mitochondria, mutations, thiol peroxidase, yeast

## Abstract

The concept that mutations cause aging phenotypes could not be directly tested previously due to inability to identify age-related mutations in somatic cells and determine their impact on organismal aging. Here, we subjected *Saccharomyces cerevisiae* to multiple rounds of replicative aging and assessed *de novo* mutations in daughters of mothers of different age. Mutations did increase with age, but their low numbers, < 1 per lifespan, excluded their causal role in aging. Structural genome changes also had no role. A mutant lacking thiol peroxidases had the mutation rate well above that of wild-type cells, but this did not correspond to the aging pattern, as old wild-type cells with few or no mutations were dying, whereas young mutant cells with many more mutations continued dividing. In addition, wild-type cells lost mitochondrial DNA during aging, whereas shorter-lived mutant cells preserved it, excluding a causal role of mitochondrial mutations in aging. Thus, DNA mutations do not cause aging in yeast. These findings may apply to other damage types, suggesting a causal role of cumulative damage, as opposed to individual damage types, in organismal aging.

## Introduction

The nature of aging remains one of the grand mysteries of biology (*Kirkwood & Austad,* 2000). In the 1950s, Failla and Szilard proposed that random somatic mutations cause cell and tissue dysfunction, leading to aging (Failla, 1958; Szilard, 1959). Maynard Smith questioned this hypothesis (Maynard, 1959), initiating the debate that is not resolved to this day. Previous studies revealed accumulation of age-related mutations in human cells (Oller *et al*., 1989; Jones *et al*., 1995), mice (Dolle *et al*., 2000), and flies (Garcia *et al*., 2010). However, the molecular mechanisms involved remain unknown. One possibility is that decreased fidelity (e.g., due to protein damage) of enzymes responsible for preventing or repairing DNA damage leads to mutation accumulation and aging (Andressoo *et al*., 2006; Brosh & Bohr, 2007; Gorbunova *et al*., 2007), but this could also be due to critical mutations in these genes accumulated through the lifespan. Deficiency in these enzymes can lead to premature aging, but whether they limit lifespan under conditions of their sufficiency is unclear. Another possibility is the accumulation of oxidative damage, which decreases or abolishes the activities of repair enzymes. Oxidative stress is considered as one of the main factors in the development and progression of cancer and other age-related diseases (Ames *et al*., 1993).

While previous data are consistent with increased mutation load as a function of age, it remains unclear whether mutations in the DNA, the only theoretically nonrenewable molecular species within cells (the species that can be modified irreversibly), cause aging. This is because the concept that mutations cause functional decline and other aging phenotypes could not be directly tested due to inability to fully assess age-related mutations in individual somatic cells and determine their impact on organismal aging. Recently, single-cell sequencing technology has been applied to individual normal human clonal crypt, showing that chromosomal rearrangements occur in the last decades of normal human lifespan (Hsieh *et al*., 2013). However, it has been hard to determine if these mutations contribute to the aging process.

In this regard, the unicellular yeast, *Saccharomyces cerevisiae*, could be very useful in addressing the half-century-old debate. Yeast cells produce a certain number of daughter cells before they die, and the total number of daughter cells produced by the mother defines her replicative lifespan (Kaeberlein *et al*., 2007). Significant conservation of proteins in the DNA repair pathway between yeast and higher eukaryotes further elevates the yeast as a model to study age-related genomic instability (Bitterman *et al*., 2003). Most importantly, the yeast genome is well characterized, and individual cells can give rise to colonies that can be sequenced, thereby establishing the genotype of the daughter. Moreover, the yeast genome is small, so the impact of its mutations can be quantified, which is more difficult to do in the case of model organisms with large genomes. Here, we subjected *S. cerevisiae* to multiple rounds of replicative aging and assessed *de novo* mutations in daughters of mothers of different age. The data show that DNA mutations do not cause aging in yeast.

## Results and discussion

**Fig 1:**
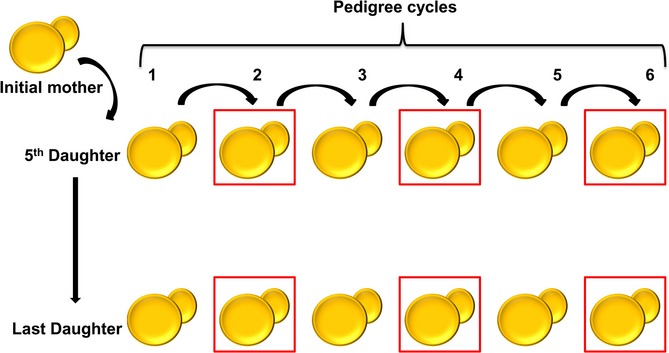
Experimental design of the pedigree assay. Virgin mothers (shown as initial mother in the figure) were subjected individually to replicative lifespan, and their 5th and last daughters isolated to give rise to individual clones. Virgin mothers from these clones were again subjected to the replicative lifespan procedure, isolating either 5th (from the ‘young’ lineage) or last (from the ‘old’ lineage) daughters. This procedure was repeated six times for four independent WT and four independent Δ8 lines, followed by genome sequencing of the respective colonies following the 2nd, 4th, and 6th cycles (shown by red squares). In total, 48 genomes of individual cell-derived lineages were sequenced and analyzed.

**Fig 2:**
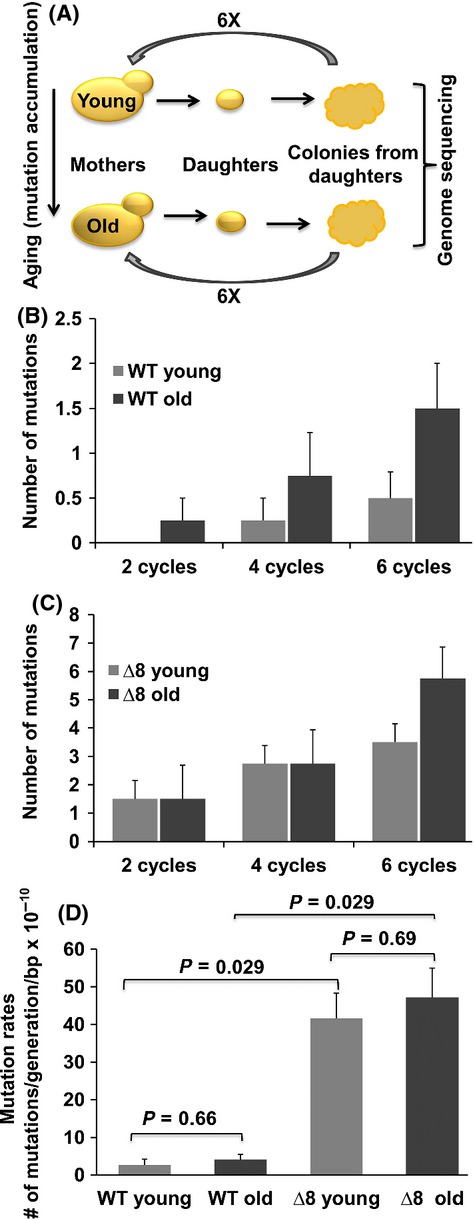
Mutation accumulation does not cause aging in yeast. (A) Overall experimental design of the study. Individual yeast cells (mothers) were subjected to replicative aging, and their daughters were collected at different ages of mother cells. These cells gave rise to colonies, from which new mother cells were taken and subjected to a second round of the aging procedure. The genomes of young and old lines after 2, 4, and 6 cycles were sequenced. See Fig.1 for details. (B) Average number of mutations per aging cycle of young and old WT cells. (C) Average number of mutations per aging cycle of young and old Δ8 cells. Error bars represent the standard error of mean. (D) Mean mutation rate (number of mutations per line divided by the number of cell divisions and the length of the genome). The graph shows the mean of four lines in each experiment, and error bars are standard errors. *P*-values for each comparison were calculated using the Wilcoxon rank-sum test.

To address the role of DNA mutations in aging, we subjected *Saccharomyces cerevisiae* to replicative lifespan analyses and sequenced the genomes of clones derived from individual daughter cells of mothers of different age. First, we collected 5th (designated young) and last alive (∼30th, designated old) daughter cells from each of the 4 wild-type (WT) mother cells and generated colonies from them (Figs1 and 2A). We then used virgin cells from these colonies as mothers and again collected 5th (from the young lineage) and last (from the old lineage) daughters and made colonies from them. This procedure was repeated six times by selecting young cells from young lineages and old cells from old lineages (Figs1 and S1), followed by high-coverage sequencing of the genomes (>99.99% completeness) of single cell-derived colonies after 2, 4, and 6 cycles of the procedure (a total of 24 genomes).

Analyses of these nuclear genomes revealed a steady increase in *de novo* mutations (base substitutions and indels) (Table1) with each cycle of the procedure (Fig.2B). After six cycles, young cells accumulated ∼0.5 mutations/cell and mutations increased threefold in old cells (*P* < 0.03), consistent with the previous data in flies and mice, representing both dividing (mouse) and postmitotic (fruit fly) cells (Dolle *et al*., 2000; Garcia *et al*., 2010). However, the low number of mutations observed in old yeast cells, ∼ 1.5 mutations per six cycles or ∼ 0.4 mutations per lifespan of an average cell, was inconsistent with the causal role of these mutations in aging. This finding also agrees with observations of yeast lifespan. For example, although daughters of very old mother cells have a shorter lifespan, the granddaughters restored lifespan to that observed in the daughters of young mothers (Kennedy *et al*., 1994). We also found that the lifespan of progeny of young and old cells at the end of six cycles was similar and matched that of original cells (Fig. S2). In addition, a recent report demonstrated that spores generated by aging diploid cells had the same replicative potential as those from young cells (Unal *et al*., 2011). Thus, while nuclear mutations do accumulate with age, they do not cause aging.

**Table 1:** Mutations observed in young and old WT clones following pedigree analysis

	WT							
	Y1	Y2	Y3	Y4	O1	O2	O3	O4
Substitutions Position								
Noncoding	0	0	1	0	1	0	0	1
Nonsense	0	0	0	0	0	0	0	0
Missense	0	0	0	0	0	0	1	0
Silent	0	0	0	0	0	0	0	0
Indels Position								
Noncoding	1	0	0	0	0	1	0	2
Coding	0	0	0	0	0	0	0	0
All Mutations	1	0	1	0	1	1	1	3
Mean (×10^−10^)	2.65 × 10^−10^				4.1 × 10^−10^			

Mutation distributions for each pedigree lines are shown after completion of six cycles of aging. Mean mutation rates are calculated based on the total number of substitutions and small indels divided by the total number of generations for four lines. The whole list of mutations together with positions and nucleotide changes is shown in Table S1. Y is young, and O is old.

Our further analysis of the observed mutations revealed that many of them occurred on chromosome XII, where the ribosomal DNA (rDNA) repeats are located. rDNA has been identified as a factor leading to ERCs, which accumulate during replicative aging (Sinclair & Guarente, 1997). It was also shown that rDNA instability is a contributing factor to the aging process (Kobayashi, 2008) and that aging is accompanied by a significant increase in loss of heterozygosity (Lindstrom *et al*., 2011). In this regard, our observation of a higher mutation rate in this region is consistent with previous findings. An increased number of rDNA reads was recently shown in aged mother cells (Hu *et al*., 2014). We also analyzed the reads aligned to the rDNA locus in both young and old cells and found no large deletions/insertions or an increase in the number of rDNA reads during replicative aging (Fig. S3).

To assess the contribution of oxidative stress to mutation accumulation and compare it with age-dependent mutation load, we similarly analyzed a mutant strain (designated Δ8) lacking eight thiol peroxidases (Fomenko *et al*., 2011; Kaya *et al*., 2014), which are the enzymes (peroxiredoxins and glutathione peroxidases) that detoxify hydroperoxides. We further sequenced the 24 genomes corresponding to young and old Δ8 cells after 2, 4, and 6 cycles, similarly to the procedure described above for WT cells (Table2; Figs S1 and S4). This mutant showed a 12-fold higher mutation rate than WT cells, ∼ 6 mutations per six cycles (Fig.2C). However, even young Δ8 cells had twice as many mutations as the old WT cells, and this situation did not correspond to the aging patterns of these cells, that is, old WT cells had few mutations, yet stopped dividing and died, whereas young Δ8 cells had many more mutations, yet they continued dividing. The overall substitution mutation rate of WT cells was in line with the known mutation rate in yeast (Lynch *et al*., 2008) (Fig.2D). Although larger sample sizes would have allowed measuring the mutation rates more precisely, our goal was to determine the actual number of mutations in individual yeast cells per lifespan. It was clear from our analysis that both young and old WT cells had a much lower mutation rate than either young or old Δ8 cells (Fig.2D). We also examined mutations in repetitive regions, which are thought to exhibit higher mutation rates (Lynch *et al*., 2008) and impact protein quality control (Nyström & Liu, 2013), but detected no such mutations during aging. Together, these findings, and the low number of mutations in old cells (< 1 per lifespan), exclude a causal role of mutation accumulation in aging in yeast.

**Table 2:** Mutations observed in young and old Δ8 clones following pedigree analysis

	Δ8							
	Y1	Y2	Y3	Y4	O1	O2	O3	O4
Substitutions Position								
Noncoding	0	0	0	1	4	3	1	0
Nonsense	0	0	0	0	0	0	0	0
Missense	1	1	1	0	2	0	1	0
Silent	1	3	1	0	1	1	1	1
Indels Position								
Noncoding	1	1	2	1	0	1	1	1
Coding	0	0	0	0	1	0	3	1
All Mutations	3	5	4	2	8	5	7	3
Mean (×10^−10^)	41.5 × 10^−10^				47.1 × 10^−10^			

Mutation distributions for each pedigree lines are shown after completion of six cycles of aging. Mean mutation rates are calculated based on the total number of substitutions and small indels divided by the total number of generations for four lines. The whole list of mutations together with positions and nucleotide changes is shown in Table S2. Y is young, and O is old.

**Fig 3:**
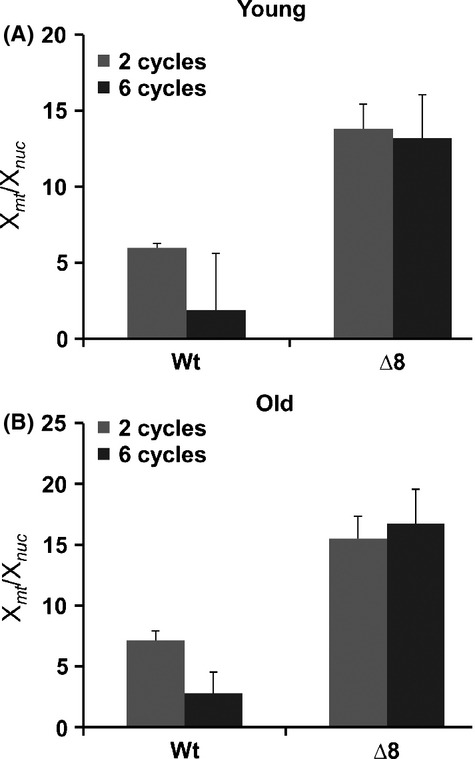
Mitochondrial content is higher in Δ8 cells and does not decrease during aging. Genomes of individual clones (four young and four old at each cycle) were sequenced following 2, 4, and 6 cycles of aging. *X*_mt_ coverage (mitochondrial genome) divided by *X*_nuc_ coverage (nuclear genome) is shown. (A) Mitochondrial genome abundance of young WT and Δ8 cells following 2 and 6 cycles. (B) Mitochondrial genome abundance of old WT and Δ8 cells following 2 and 6 cycles.

Do mutations in the yeast mitochondrial genome cause aging? As mitochondria are the major source of reactive oxygen species, mtDNA may be vulnerable to oxidative damage (Harman, 1956). The mitochondrial genome has a higher mutation rate than the nuclear genome (Lynch *et al*., 2008), and the biology of yeast mitochondria has been implicated in aging (Breitenbach *et al*., 2012). We found that mtDNA was consistently lost in WT cells after they generated 10–20 daughter cells, and this effect was also seen in young cells after several aging cycles, making many of these cells respiration deficient (Fig.3; Figs S5 and S7). The observation that cells lose mtDNA during the replicative aging process leading to nonrespiring cells was also reported previously (Veatch *et al*., 2009; Lindstrom *et al*., 2011). An interesting observation was that some of these cells showed mitochondrial mutations just prior to completely losing the mitochondrial genome, explaining the loss of the mitochondrial genome during the aging progress. Although yeast cells grown on glucose do not rely on respiration and do not utilize mtDNA, mutations may disrupt integrity of mtDNA, so it may be lost. Interestingly, among the cells that we analyzed, the two old WT lines that still had functional mitochondria possessed 10 and 6 distinct noncoding substitution mutations.

In contrast, Δ8 cells had a higher mtDNA content in both old and young cells, did not lose mtDNA during aging and progression through aging cycles, and did not accumulate mutations in the mitochondrial genome (Fig.3; Figs S6 and S7). Although in some samples we observed heterogeneous mutations (i.e., both original and mutant sequences were present) none of them persisted, that is, mutated mtDNA was lost in subsequent cycles, whereas the original mtDNA remained. Throughout the procedure, Δ8 cells were fully dependent on respiration, yet their lifespan was shorter than that of WT cells. Thus, not only mtDNA mutations, but even the presence of the functional mitochondrial genome, did not correlate with the aging process. A possibility remains that transient heterogeneous mitochondrial mutations in Δ8 cells contribute to the aging process at the level of individual mitochondria, but deleterious mutations are selected against during colony expansion. It should also be noted that although an increase in oxidative stress levels leads to increased mutation load, oxidative stress also targets numerous other molecules. It is unlikely that these conditions affect only DNA in Δ8 cells. In future studies, it would be useful to use a strain, which has a mutated proofreading domain of a nuclear DNA polymerase, thus leading specifically to increased mutation load without affecting other cellular systems.

It would also be interesting to understand why Δ8 cells rely on respiration. It is known that age asymmetry between the mother and daughter cells is affected by the end of life of mother cells (McMurray & Gottschling, 2004). Old cells pass some of the damaged molecules including aggregated proteins and dysfunctional mitochondria to their daughter cells (Henderson & Gottschling, 2008; Steinkraus *et al*., 2008; Kaeberlein, 2010), and daughters of old mothers live a shorter life (Kennedy *et al*., 1994). However, granddaughters of old mother cells are apparently able to clear the damage (McMurray & Gottschling, 2004; Kaeberlein, 2010). Previously, age-dependent deterioration in mitochondrial genome integrity was shown in mice; however, mitochondrial mutations did not limit the lifespan of these animals (Vermulst *et al*., 2007). In yeast, it was also shown that the absence of the mitochondrial genome does not affect the replicative lifespan in both BY4742 and YDW2 backgrounds (Kaeberlein *et al*., 2005; Woo & Poyton, 2009), and the replicative lifespan is increased in JM43 and D273-10B backgrounds (Woo & Poyton, 2009).

Aging is often viewed in terms of gradual degeneration and accumulation of damage, leading to decline in fitness. We agree with this notion of aging as a gradual process driven by damage (in the broad sense of it, that is, damage not limited to by-products and errors) (Gladyshev, 2013). Universality of the aging process and the conservation of aging pathways and genetic and dietary interventions that affect lifespan point to a common basis for aging in diverse organisms. Point mutations represent an important form of cellular damage, but not the only one. For example, genome rearrangements have been shown to increase during replicative (McMurray & Gottschling, 2004) and chronological (Longo & Fabrizio, 2012) aging in yeast. However, we found no structural genome rearrangements during aging in the cells that we examined. A possibility remains that point mutations and structural rearrangements are eliminated during cell growth, that is, they accumulate in large numbers during aging, but selection acts during colony expansion of daughters of old cells eliminating them. However, we think it is highly unlikely that all mutations and rearrangements can be eliminated, returning the genome to the original state, especially considering that most age-related mutations are expected to be neutral.

A recent study analyzed populations of yeast old mother cells, in which more than ∼ 85% of cells ceased to divide, and observed many genomic rearrangements accompanying aging, including mitochondrial DNA transfer to the nuclear genome, translocations, and retrotranspositions (Hu *et al*., 2014). This study cannot be directly related to our conclusion that DNA mutations do not cause aging (but together the two studies hold significant information about progression of replicative aging and the processes after terminally old mother cells stop dividing). This is because that other study analyzed enriched, purified aged mother cells at the population level, whereas we did this at the individual cell level, which was required to assess the causal role of mutations. It is possible that a significant fraction of the genomic rearrangements observed (Hu *et al*., 2014) occurred after the last daughter was formed, or these mutations occurred in a fraction of dying cells in the population. Thus, we cannot exclude a possibility that mutations contribute to demise of the mother cell (which is synonymous with her inability to bud off one more daughter). But, this would be equivalent to the contribution of mutations to death of an organism, as opposed to having a causal role in the aging process.

Another recent study analyzed Ty1 retrotransposon expression and mobility during chronological aging in *S. paradoxus* (VanHoute & Maxwell, 2014). Increased expression and mobility of retrotransposons with age have been found in nearly every aging model and thought to promote genome instability during aging (Moskalev *et al*., 2013). However, this study showed that, while the strain expressing Ty1 element had 40-fold increased mobility to the rDNA region, neither Ty1 mobility nor increased mutation rate of a representative Ty1 gene led to chronological lifespan decrease. Furthermore, increased expression of a Ty1 could extend chronological lifespan under certain conditions (VanHoute & Maxwell, 2014).

Replicative aging in yeast can be defined as the progression of mitotic division until an ultimate phenotype that prevents further daughter cell formation. During progression of aging, accumulation of different types of damage has been reported. If a single damage form limits lifespan, selection on other damage forms should be relaxed, leading to a certain degree of synchronization with regard to their deleterious impact. Thus, we speculate that application of our findings to other individual damage types and to other organisms suggests that there is no individual damage form that represents, when considered in isolation, a main causal factor in aging. If a damage form decreases fitness, organism can develop protective strategies to downgrade it to a milder form or modify its metabolism to decrease production of this damage. But the mild damage forms are too numerous to be protected against (Gladyshev, 2013). Thus, even if mutations and other individual damage forms, when taken in isolation, do not cause aging, aging may still result from cumulative damage, to which these damage forms contribute. From this perspective, mutations in nuclear and mitochondrial genomes, together with the myriad of other damage forms, contribute to normal aging, but can cause aging only at the level of cumulative damage.

## Experimental procedures

### Yeast strains

All yeast strains were in BY4741 background. A mutant strain, lacking all eight thiol peroxidases, was reported previously (Fomenko *et al*., 2011). Cells were grown on yeast extract–peptone–dextrose (YPD) medium.

### Pedigree analyses and mutation detection

WT and Δ8 cells were grown at 30°C for 2 days. Several virgin mothers were placed in predetermined positions with a micromanipulator. We isolated 5th and last daughters from these mothers and allowed them to form colonies. For the 2nd round, single virgin cells from these colonies were subjected to replicative lifespan analysis, and 5th (from each young line) and last (from each old line) daughters were collected again, to obtain additional colonies. This procedure was repeated six times. At the end of the 6th cycle, DNA was isolated from each colony and paired-end sequenced on an Illumina platform (400 bp insert size, 75 bp nucleotide reads, paired end). The depth of coverage for each line was between 50X and 150X. Burrows–Wheeler Aligner (BWA) was used for alignment (Li & Durbin, 2009), revealing more than 99.99% genome completeness (regions covered by more than three reads). Pairwise comparisons of sequenced genomes were performed using the Genome Analysis Toolkit (GATK) (McKenna *et al*., 2010) to identify mutations covered by at least three reads and with the GATK quality score higher than 10 (*P* = 0.001 for misidentification). The data were further analyzed using the SHORE toolkit (Schneeberger *et al*., 2009), the nucleotide variants were cross-checked with the GATK output, and mutations were manually verified with Tablet (Milne *et al*., 2010). We also adjusted parameters to include cases in which the fraction of reads supporting an alternative allele was lowered to 0.5; however, this did not result in an increase in the number of candidate mutations. In addition, variable tandem repeats were extracted from Vinces *et al*., 2009 (intergenic repeats) and Verstrepen *et al*., 2005 (intragenic repeats) and further subjected to our analysis.

### Calculation of mutation rates

Mutation rate was calculated as the number of mutations per line divided by the number of cell divisions and the length of the genome. The figures show the mean of four lines in each experiment, and error bars are standard errors. *P*-values for each comparison were calculated using the Wilcoxon rank-sum test.

### Analyses of mitochondrial mutations and the rDNA locus

For normalization, *X*_mt_ coverage (mitochondrial genome) was divided by *X*_nuc_ coverage (nuclear genome). The same procedure was also applied to analyze the rDNA locus (*X*_rDNA_). The total number of reads mapped to the rDNA locus was divided by *X*_nuc_ coverage and plotted. YPEG plates containing ethanol and glycerol were used to assess growth on respiratory substrates. Petite colony formation was performed as previously described (Ferguson & Von Borstel, 1992). Antimycin A sensitivity was tested on YPD plates containing 2 μg/mL of antimycin A.

## Supporting Information

**Fig. S1** Age at which daughter cells of young and old WT mothers were taken for further analysis. **Fig. S2** Lifespan of clones derived from young and old mother cells is not affected by the aging process. **Fig. S3** Comparison of the reads mapped to the rDNA region of WT young and old lines. **Fig. S4** Age at which daughter cells of young and old Δ8 mothers were taken for further analysis. **Fig. S5** Depletion of mitochondrial DNA and respiratory function in WT lines during aging. **Fig. S6** No depletion of mitochondrial DNA in Δ8 lines during aging. **Fig. S7** Induction of petite colony formation. **Table S1** Mutations identified in WT cells during aging. **Table S2** Mutations identified in Δ8 cells during aging.
