# Supplementary material for: Evidence that mutation accumulation does not cause aging in Saccharomyces cerevisiae
Source: Aging Cell. 2015 Feb 22;14(3):366–71. doi: 10.1111/acel.12290 (PMC4406665; doi:10.1111/acel.12290)
Supplement: Supplementary file 1 [file acel0014-0366-sd1.pdf]

Table S1. Mutations identified in WT cells during aging.

WT Young				
Chromosome	Location	Type of mutation	Change	Coding/Noncoding
Chr_11	666557	–	T	
Chr_12	732284	#	A/G	

WT Old				
Chr_2	263159	–	G	
Chr_12	97258	#	G/A	SSA2
Chr_12	520616	-	T	
Chr_12	734685	+	C	
Chr_14	61678	#	A/C	
Chr_15	727926	#	G/A	NOC2

The table shows deletions (-), insertions (+), and base substitutions (#), together with chromosome localization and nucleotide changes, identified in WT cells. If a mutation is in the coding region, gene name is indicated on the right. It should be noted that half of the mutations were detected on chromosome 12, which also houses sequences coding for ribosomal RNA. However, none of these mutations were recurrent, and chromosome 12 mutations were not overrepresented in Δ8 cells during aging (Table S2).

Figure S1

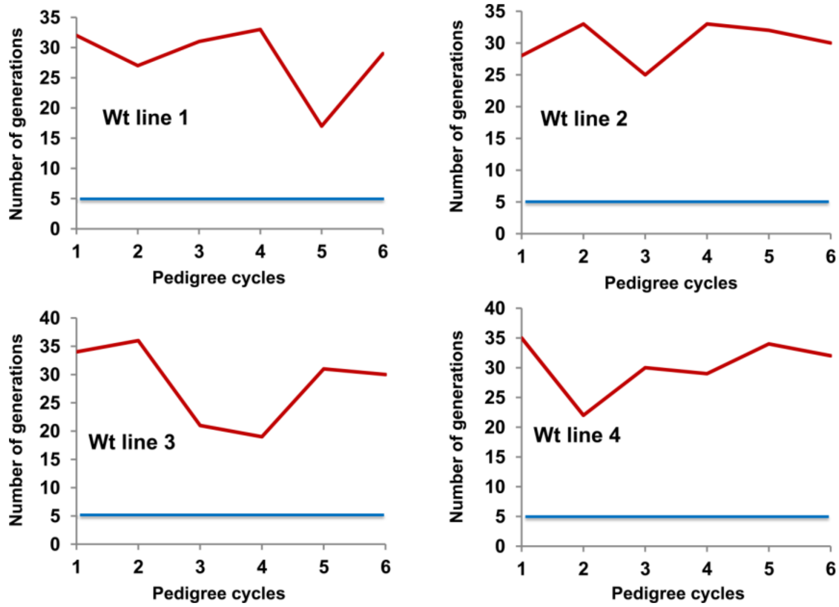

Figure S1. Age at which daughter cells of young and old WT mothers were taken for further analysis. Four lines of young (blue) and four lines of old (red) WT cells were subjected to the pedigree analysis. The age at which cells were obtained for genome sequencing for young (always 5th daughters) and old (last daughters; varies depending on the age at which a mother produced its last daughter) clones is shown.

Table S2. Mutations identified in ∆8 cells during aging.

Chromosome	Location	Type of mutation	Change	Coding/Noncoding
**∆8 Young**				
Chr_4	384317	−	C	
Chr_4	688062	+	TT	
Chr_4	834281	#	G/C	YDR186C
Chr_4	1518948	+	T	
Chr_7	1070941	#	G/A	MAL13
Chr_8	2292	+	C	
Chr_8	85398	+	A	
Chr_10	467499	#	G/A	PTE1
Chr_12	406250	#	C/A	ACE2
Chr_12	519717	#	A/G	SWI6
Chr_12	1030825	#	G/C	
Chr_13	752348	#	G/T	YMH2
Chr_15	291728	#	T/C	TLG2
Chr_16	648348	#	A/C	TIP41
**∆8 Old**				
Chr_2	101155	−	G	
Chr_2	299554	−	C	RKM3
Chr_2	497096	#	A/G	
Chr_4	384299	#	C/G	
Chr_4	623559	#	C/G	YDR089W
Chr_4	711973	#	G/A	MTC5
Chr_4	805923	#	G/A	
Chr_5	240143	+	T	
Chr_6	106277	−	TT	
Chr_6	119038	#	A/T	
Chr_7	720393	−	C	
Chr_7	987105	−	T	
Chr_8	94651	#	G/T	
Chr_10	523983	#	G/A	
Chr_12	228270	#	A/T	RIC1
Chr_12	346457	+	TG	
Chr_12	445674	#	C/A	ACE2
Chr_14	117087	#	C/T	
Chr_14	784143	−	G	
Chr_15	553600	#	C/A	LEO1
Chr_15	631626	#	C/T	PUP1
Chr_16	261384	#	A/G	
Chr_16	698035	#	T/A	YPR078C

Deletions (-), insertions (+), and base substitutions (#), together with chromosome localization and nucleotide changes, identified in ∆8 cells. If a mutation is in the coding region, gene name is shown on the right.

Figure S2

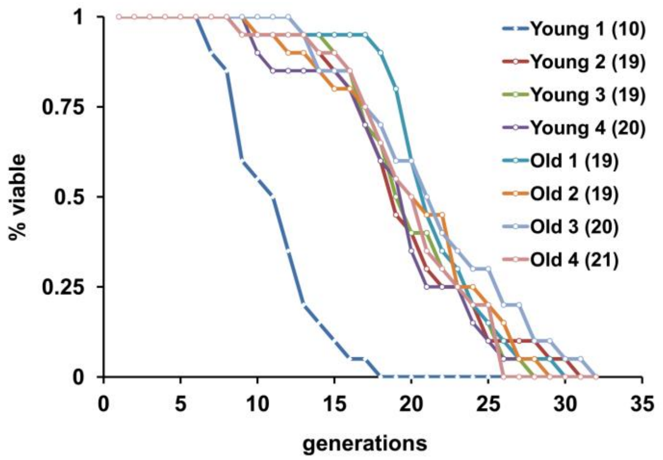

Figure S2. Lifespan of clones derived from young and old mother cells is not affected by the aging process. Replicative lifespan analysis of young and old WT cells following 6 cycles of aging is shown. Lifespan was analyzed for WT cells on standard glucose (2%) medium. Mean lifespan is shown in parentheses for each line.

Figure S3

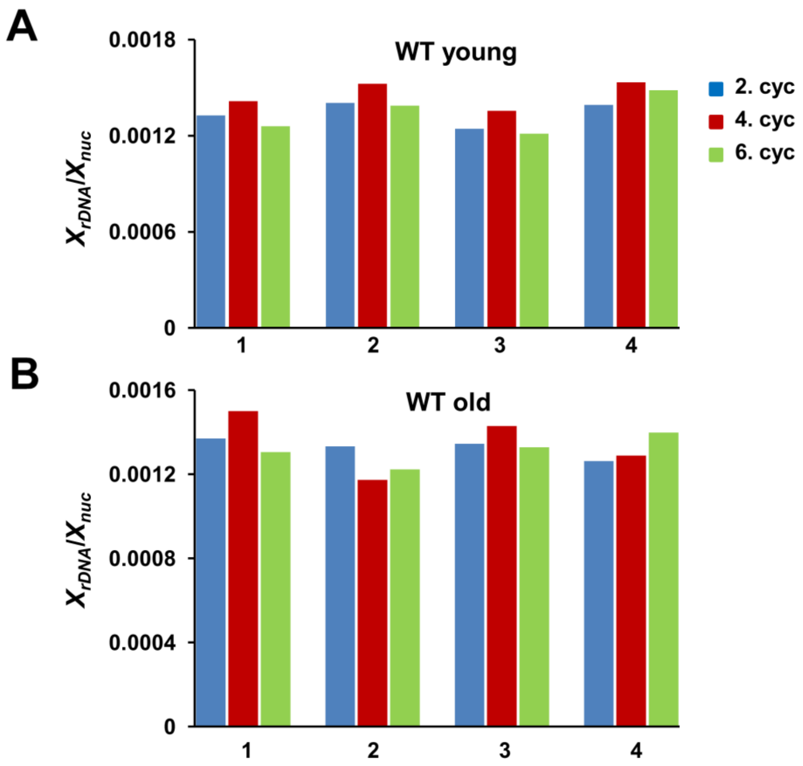

Figure S3. Comparison of reads mapped to the rDNA region of young and old WT lines. Normalized rDNA coverage per aging cycle for each of the four A) young and B) old WT lines (indicated by numbers 1, 2, 3 and 4) is shown.

Figure S4

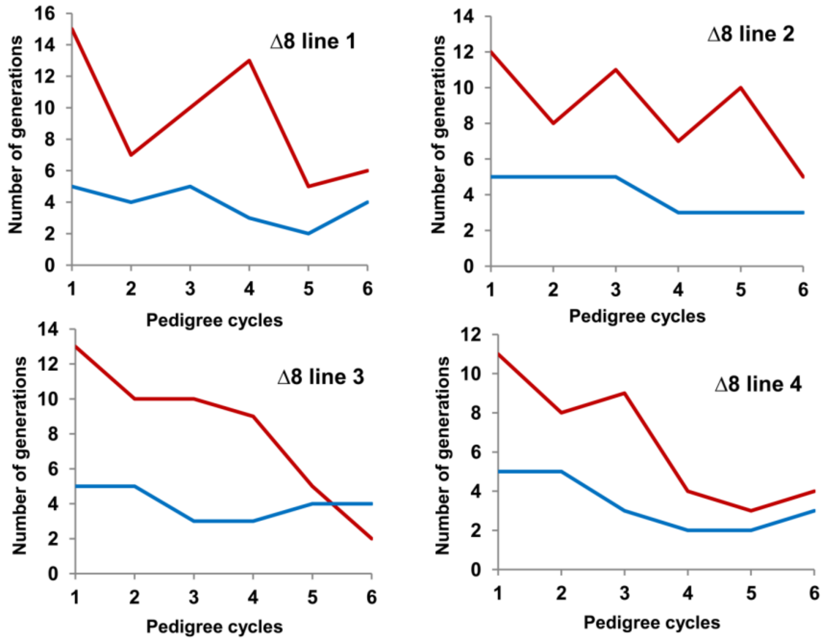

Figure S4. Age at which daughter cells of young and old Δ8 mothers were taken for further analysis. Four independent lines of young (blue) and four independent lines of old (red) Δ8 cells were subjected to the pedigree analysis. The age at which cells were obtained for young (initially 5th daughters; the age varied because some mother cells died earlier than 5 generations) and old (last daughters; varied depending on the age at which a mother produced its last daughter) clones is shown.

Figure S5

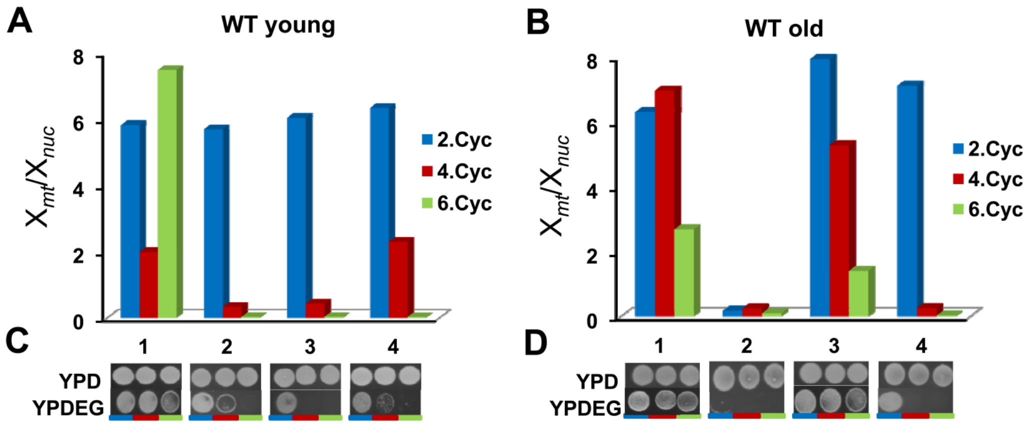

Figure S5. Depletion of mitochondrial DNA and respiratory function in WT lines during aging. Normalized mtDNA coverage per aging cycle for each of the four A) young and B) old WT lines (indicated by numbers 1, 2, 3 and 4) is shown. The young (C) and old (D) clones from each aging cycle were tested for respiratory growth on YPD (as control) and YPDEG (containing ethanol and glycerol as carbon sources) plates.

Figure S6

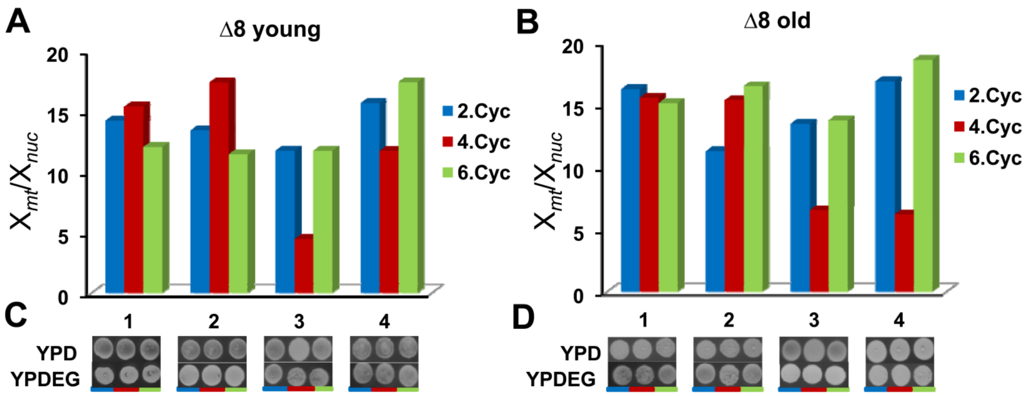

Figure S6. No depletion of mitochondrial DNA in Δ8 lines during aging. Normalized mtDNA coverage per aging cycle for each of the four A) young and B) old Δ8 lines (indicated by numbers 1, 2, 3 and 4) is shown. The young (C) and old (D) clones from each aging cycle were tested for respiratory growth on YPD (as control) and YPDEG (containing ethanol and glycerol as carbon sources) plates.

Figure S7

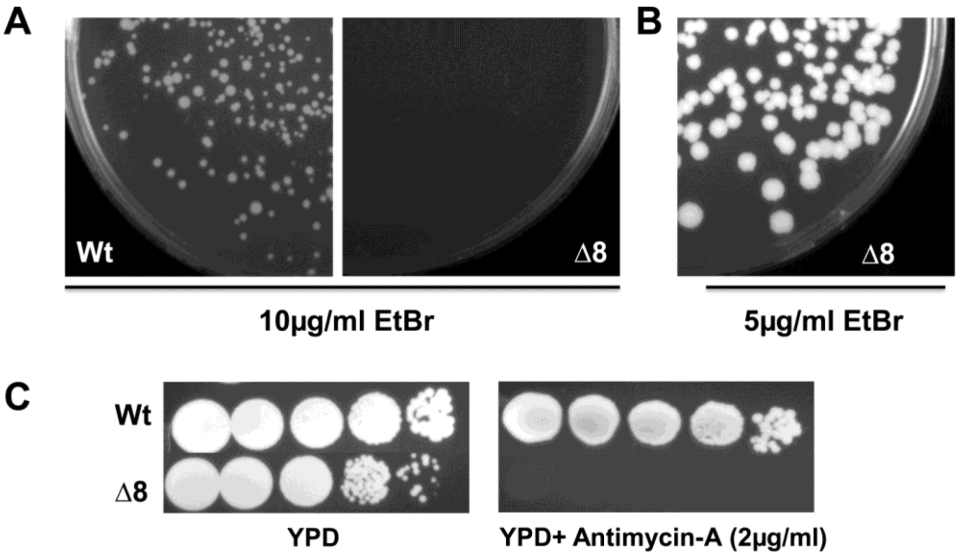

Figure S7. Induction of petite colony formation. A) WT and Δ8 cells were treated with 10 µg/ml EtBr to induce *rho*^{0} formation. While WT cells survived, Δ8 cells required mitochondrial function for viability. B) Low dose (5 µg/ml) treatment of EtBr did not induce petite colony in Δ8 cells. C) Spot assay for measurement of Antimycin-A sensitivity. The data show that Δ8 cells do not withstand the treatment, whereas no affect was observed for WT cells.
